# Origins of allostery in vertebrate hemoglobin evolution

**DOI:** 10.64898/2026.05.25.727495

**Published:** 2026-05-26

**Authors:** Carlos R. Cortez-Romero, Naim M. Bautista, Collin R. Nisler, Ricardo Muñiz-Trejo, Jay F. Storz, Joseph W. Thornton

**Affiliations:** 1 Program in Cell and Molecular Biology, University of Chicago; Chicago, 60637, USA.; 2 Department of Chemistry, University of Chicago; Chicago, 60637, USA.; 3 Department of Ecology and Evolution, University of Chicago; Chicago, 60637, USA.; 4 Department of Human Genetics, University of Chicago; Chicago, 60637, USA.; 5 School of Biological Sciences, University of Nebraska; Lincoln, 68058, USA.

## Abstract

Allostery—regulation of a protein’s activity by binding to an effector—is an essential functional feature of many proteins. Its structural basis is complex: a protein must bind effector at one site—an interaction that typically involves many complementary residues—and this binding event must change the protein’s conformation at the distant active site. How allosteric proteins evolved this architecture is unknown because there are no cases in which the historical mechanisms by which allostery was acquired from nonallosteric precursors have been identified. Vertebrate hemoglobin (Hb) is a tetrameric protein whose oxygen affinity is reduced by binding organic phosphates. We used ancestral protein reconstruction, biochemical experiments, and in silico studies of protein structure and dynamics to identify the changes in protein sequence and structure that caused allosteric Hb to evolve from its non-allosteric dimeric precursor. We found that just two historical substitutions were sufficient to confer positive allostery on the ancestor—one that caused the dimer to tetramerize, and another that created an effector binding site in the tetramer’s central cavity. Two additional substitutions changed the effector’s position and conferred the modern form of negative allostery. This short evolutionary path to allostery was possible because most of the key requirements for allostery already existed as by-products of the protein’s structure: the tertiary transition between oxygenated and deoxygenated conformations is an ancient and intrinsic property of the globin fold. It affects the surface patch that ultimately mediated tetramer assembly, so as soon as tetramerization evolved because of one substitution on that surface, tertiary heterogeneity propagated to large-scale quaternary heterogeneity. The other substitution(s) conferred effector binding at a site within the tetramer that is preferentially accessible in the deoxygenated state. One of the most complex and essential protein phenomena therefore evolved via simple and intelligible mechanisms.

## Introduction

Allostery –modulation of a protein’s activity by binding to an effector molecule or other reversible post-translational modification – is a critical mode of biochemical and cellular regulation across the tree of life and has therefore been called “the second secret of life.” (1, 2). How this complex property evolved from non-allosteric precursors is poorly understood.

The mechanisms within extant proteins that mediate allostery are well established (3–6). Allostery requires a protein to meet several necessary and sufficient conditions: it must be capable of occupying distinct conformations that differ in their functional activity, and it must bind effector with affinity that differs between these conformations (5, 7, 8). How these properties evolve from non-allosteric precursors is unknown. Each feature involves numerous residues in a protein, so one might expect that the evolution of allostery from a non-allosteric precursor would require many mutations. But it is also conceivable that nonallosteric proteins may already possess some of the required properties; this would allow allostery to evolve if mutations were to confer the remaining prerequisites, as observed in some biochemical studies (9–12).

A major challenge in understanding the historical origins of allostery is that the causes of ancient evolutionary changes in protein properties are often obscured or elaborated by subsequent modifications of sequence and structure (13). This challenge can be addressed by experimentally introducing historical sequence substitutions into phylogenetically reconstructed and synthesized ancestral proteins (13, 14). A few studies have used this strategy to understand how already-allosteric proteins evolved specificity for new effectors and how the functional impact of effector binding in allosteric proteins may switch from positive to negative regulation (15–17). But no work to date has addressed how any protein acquired effector-driven allostery from a nonallosteric precursor (18).

**Fig. 1.**
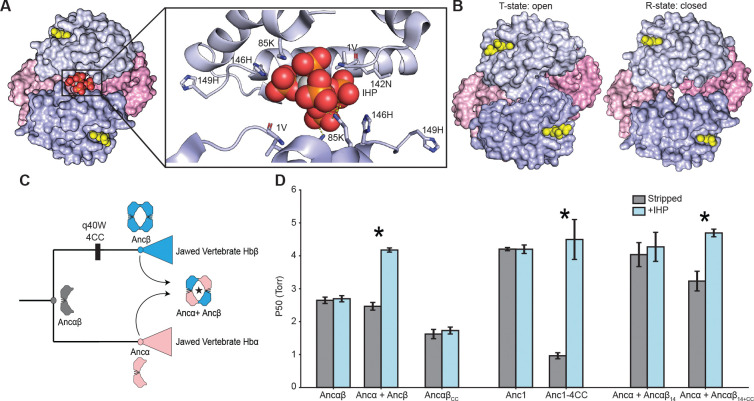
Four stubstitutions confer allostery in the ancestral homotetramer. **(A)** Crystal structure of Human Hb bound to IHP (PBD:3HXN). Blue, β subunits. Pink, ɑ subunits. Yellow spheres, heme. IHP is shown as spheres colored by element (red, oxygen; orange; phosphate; gray, carbons. Inset, the central cavity with IHP bound, with residue that interact with IHP shown as sticks. **(B)** *Left:* Hemoglobin tetramer central cavity is open when the hemes are deoxygenated (PDB:4HHB). *Right:* Hemoglobin central cavity is closed when hemes are bound to oxygen (PDB:2DN1). **(C)** Evolution of allosteric regulation on the HB phylogeny. Icons, oligomeric states determined by experimental characterization of reconstructed ancestral proteins. **(D)** Oxygen affinity of Ancαβ variants in the presence and absence of IHP, shown as the partial pressure of oxygen at which half-maximal binding is achieved. Gray, stripped condition in which IHP is absent. Blue, 500 μM of IHP added to solution. Error bars, standard error of measurement, n = 5.

Here we applied this strategy to understand the evolutionary origin of effector-driven allostery in vertebrate hemoglobin (Hb), the major oxygen carrier in the blood of jawed vertebrates. Hb is allosterically regulated by organic phosphates, such as IHP, BPG, and ATP, which reduce oxygen affinity and thereby facilitate oxygen delivery to metabolically active recipient tissues in the periphery. The structural basis for allostery in extant Hb is well understood. Hb is an α_2_β_2_ heterotetramer that can be visualized as two α_1_β_1_ heterodimers bound to each other isologously – using the same surface patch on each subunit but rotated 180 relative to each other. The tetramer binds four molecules of oxygen, one each to the heme cofactor coordinated by each globin subunits. A single effector molecule is bound in a central cavity (CC), which is formed between the two β subunits (Fig. 1A) (19–22). Hb can occupy two conformations, called T and R, which differ in oxygen affinity. In the T conformation (which has lower oxygen affinity), the two α/β heterodimers are rotated relative to the other by about 15 degrees compared to the R conformation, and the CC is open; in the higher-affinity R conformation, the CC is occluded (Fig. 1B). Effector binding is therefore associated with the T-conformation, and effector release with the R-conformation (4, 23).

When Hb’s allostery emerged during evolution has been previously established, but the mechanisms are unknown. The most closely related proteins to Hbα and Hbβ are all nonallosteric monomers, including myoglobin, the major oxygen binding molecule in muscles (24). Our previous work showed that Hb evolved from a monomeric common ancestor with myoglobin via a homodimer (Ancαβ), which was also non-allosteric and existed just before the duplication that produced the paralogous Hbα and Hbβ lineages in jawed vertebrates. Whereas Ancαβ is non-allosteric, Ancα and Ancβ– the last common ancestral proteins of all gnathostome Hbα’s and all gnathostome Hbβ’s, respectively – form a Hb-like heterotetramer that responds allosterically to organic phosphates ((24), Fig. 1A–C, figs. S1 & 2). Here we identify the genetic and biophysical mechanisms by which organic phosphate-mediated allosteric regulation was acquired in the interval after duplication of Ancαβ during the historical evolution of Hb.

## RESULTS

### 1. Four central cavity substitutions conferred allostery.

We first quantified the allosteric response that evolved in the phylogenetic interval between the Ancαβ dimer and the Ancα+Ancβ heterotetramer. We measured oxygen affinity in vitro after stripping the medium of ionic effectors and again after adding a saturating concentration of inositol hexaphosphate (IHP). Although the physiological effector in ancestral vertebrates is unknown, IHP serves as a useful and widely used experimental effector to test for Hb allostery (25–28). IHP allosterically regulates Hbs from representatives of all major vertebrate lineages, whereas other organic phosphates, such as 2,3-biphosphoglycerate (BPG), ATP, and GTP have lineage-specific effects (29–31). All these effectors regulate oxygen affinity by a similar mechanism involving reversible binding to positively charged residues in the CC via their anionic phosphate groups (22, 32, 33).

As expected, Ancαβ is non-allosteric, with an oxygen affinity of ~2.6 torr, irrespective of whether IHP is present or absent (Fig. 1D). By contrast, Ancα+Ancβ is robustly allosteric, with an oxygen affinity of 2.4 torr in the stripped condition and 4.1 torr when IHP is added (Fig. 1D). The allosteric response – a reduction in oxygen affinity associated with the effector – therefore originated during this post-duplication phylogenetic interval.

To identify the historical changes in protein sequence that conferred allostery during this interval, we identified candidate substitutions using phylogenetic and structural information. In extant Hb, the effector binds in the heterotetramer’s central cavity to a surface composed of portions of the two Hbβ subunits. We therefore reasoned that tetramerization would be necessary and that other causal substitutions may have occurred at the effector-binding site on the branch leading to Ancβ. We used as a starting point Ancαβ +q40W (Anc1), which contains a single historical substitution from this same branch that was previously shown to confer high-affinity tetramerization when introduced into the ancestral dimer Ancαβ (34). We found that Anc1 is nonallosteric: its oxygen affinity is nearly identical between stripped and IHP-added conditions, albeit weaker than that of Ancαβ, (Fig. 1D).

Four amino acid replacements occurred within the central cavity on the branch leading to Ancβ. Three of these occurred at sites that interact electrostatically with IHP’s phosphate groups (s85K, e146R, and t142S, using lower and upper cases to denote ancestral and derived states, respectively) in the crystal structure of human Hb; the fourth occurred at C-terminal residue r149H, which occupies the cavity below IHP and makes electrostatic interactions with other residues that stabilize the T conformation (Fig. 1F (35)). The substitutions at sites 85, 146, and 149 are conserved in most descendant Hbβ proteins, whereas t142S changes to other states in some descendants (fig. S3).

To test the effects of these substitutions, we introduced the derived states at all four sites into Anc1 and found that the resulting protein (Anc1–4CC) is strongly strong allosteric, with a 4-fold difference in oxygen affinity between the stripped and IHP conditions (Fig. 1D). Creating a binding site for the effector is therefore sufficient to confer allostery on the Anc1 homotetramer. As expected, tetramerization is required for allostery, because introducing 4CC into Ancαβ without the q40W substitution does not confer an allosteric response (Fig. 1D).

The evolution of allostery from a nonallosteric precursor therefore required only a few substitutions –one to confer tetramerization and at most four others at the central cavity where the effector binds. These data also show allostery could have initially evolved in a homotetramer, and that modern Hb’s dependence on the α_2_β_2_ heterotetramer for allostery was acquired later.

These conclusions are robust to several assumptions. First, Anc1–4CC is also allosterically regulated by ATP (fig. S4), indicating that its allosteric response extends to other organic phosphate effectors. Second, heteromerization evolved from the homomeric ancestor during this same interval, and we do not know the timing of this event relative to the acquisition of allostery; we therefore asked whether the same four substitutions can confer allostery when introduced into a nonallosteric ancestral heterotetramer. For this purpose, we used Ancα+Ancαβ_14_, which contains a set of 14 historical substitutions from the branch leading to Ancβ and was previously shown to assemble specifically into heterotetramers (24). We found that the Ancα + Ancαβ_14_ complex is non-allosteric, with oxygen affinity that is insensitive to IHP and is nearly identical to Anc1; introducing the 4CC substitutions into Ancαβ_14_ confers allostery on the coexpressed complex, reducing oxygen affinity significantly in the presence of IHP (Fig. 1D).

### 2. Single substitutions confer novel forms of allostery.

**Fig. 2.**
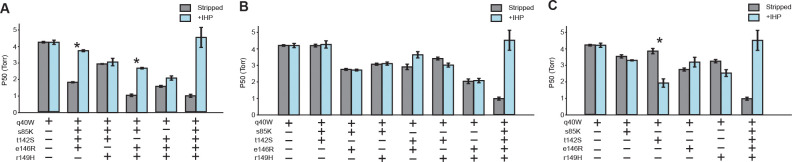
Multiple genetic mechanisms for evolving allostery. **(A)** Oxygen affinity of Anc1–4CC and triple central cavity mutants in the absence and presence of IHP at 500 μM. Error bars are standard error of measurement, n = 3. Substitutions included in each mutant are marked +; those absent as −. Asterisks, significant difference between conditions, as in Fig. 1. **(B)** All double central cavity mutants. C) All single central cavity mutants.

To understand whether all four of the central cavity substitutions were necessary to confer allostery, we created all four possible Anc1+3CC mutants by reverting each of the four cavity substitutions to its ancestral state (Fig. 2A). Two of these combinations were allosteric, with a significantly reduced affinity for oxygen in the presence of IHP, although the allosteric response is not as strong as in Anc1+4CC. Only substitution e146R was necessary for allostery, because reverting it to the ancestral state abolishes the response to IHP. This substitution and s85K, which causes the next-largest loss of allostery when reverted, both form electrostatic contacts with the effector in the human Hb crystal structure (23). Allostery can therefore be acquired by several combinations of just three historical substitutions, although all four contribute to the magnitude of the response to IHP.

We next investigated whether even fewer cavity substitutions could have conferred allostery on Anc1. One double-mutant – Anc1-s142T/e146R – has reduced oxygen affinity in the presence of IHP, but this difference was not statistically significant (Fig. 2B). Two single mutants—Anc1+s142T and Anc1+r149H—are allosteric, but the response to IHP in both cases is inverted: oxygen affinity improves rather than declines when IHP is added (Fig. 2C).

Taken together, these data indicate that the nonallosteric ancestral homotetramer Anc1 existed on the evolutionary edge of allostery. By just one to three substitutions, Anc1 can acquire either the classic form of Hb allostery by which effector binding reduces oxygen affinity or a novel form of affinity-increasing allostery that is not known in natural Hbs. Because tetramerization, which is also necessary, can be conferred on Ancαβ by q40W alone, the total number of substitutions required for the ancestral homodimer to evolve allostery is between two (for inverted allostery) and four (for the classic response).

### 3. Conformational heterogeneity pre-dated the evolution of allostery.

How can so few mutations confer allostery on Anc1? Two explanations are possible. One is that the central cavity substitutions confer all properties required for Hb allostery -- occupancy of multiple conformations, the coupling of these conformations to differential oxygen affinity, and effector binding that favors one of these conformations over another. The other possibility is that some of these properties were already present in the ancestral tetramer for reasons unrelated to allostery. The structural location of the cavity substitutions – at the site where IHP binds, but far from the active site where heme and oxygen are bound – favors the latter hypothesis: that functionally-coupled conformational heterogeneity was already present in Anc1 before allostery evolved, and the cavity substitutions conferred effector binding at a site associated more strongly with one conformation.

**Fig. 3.**
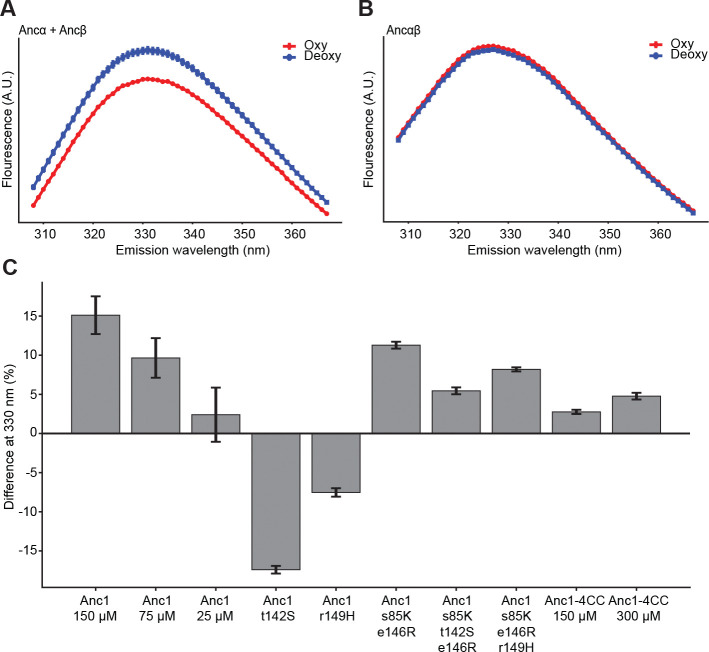
Quaternary conformational heterogeneity is present in Anc1 without allostery. **(A)** Fluorescence emissions scans of Ancα + Ancβ at 150 uM when excited at 280 nm. Red, oxygenated experimental condition. Blue, deoxygenated. Error bars, standard error of measurement, n = 10. Fluorescence is produced by exposed Trp residues; differences in quaternary structure between conditions result in differential burial of interface residue Trpp40. **(B)** Fluorescence emission scan of Ancαβ. **(C)** Difference in fluorescence emission at maximal emission wavelength between deoxygenated and oxygenated conditions. Protein concentration,150 uM, but Anc1 and Anc1–4CC were scanned at multiple concentrations to validate dependence on occupancy of the tetrameric state. Error bars, standard error of measurements, n = 10. Complete scans are in S6,7, and 9.

We tested this hypothesis using a classic intrinsic fluorescence assay of Hb’s quaternary conformation. When extant Hb binds oxygen, the rotation of the α_1_β_1_ dimer relative to the α_2_β_2_ dimer reduces the solvent exposure of residue Trp-40 at the interface between the dimers, which can be read as a reduction in fluorescence intensity at 280 nm (36–38). As expected, the allosteric tetramers human Hb and Ancα + Ancβ both display reduced fluorescence when oxygenated relative to when the protein solution is saturated with atmospheric nitrogen (Fig. 3A; fig. S5). This signature is absent in the dimer Ancαβ, which does not tetramerize and which lacks the interfacial Trp40 residue (Fig. 3B).

As predicted, the ancestral nonallosteric homotetramer Anc1 exhibits the same reduction in fluorescence signal upon oxygenation that is observed in allosteric Hbs (Fig. 3A & C, fig. S5). We observed the same oxygen-dependent change in all the central cavity mutants that display the classic form of allostery, including Anc1–4CC and the allosteric triple mutants (Fig 3C, fig. S6–9). These data show that acquisition of tetramerization is sufficient to confer the quaternary conformational heterogeneity required for allostery, and these conformations are differentially associated with the complex’s oxygen binding function. Thus, all requirements for allostery except for effector binding were already present in the Anc1 tetramer, emerging immediately upon the acquisition of tetramerization.

A different signal of quaternary change is apparent in the allosteric mutants of Anc1 that are inversely regulated by IHP. In these two proteins, the fluorimetry response to oxygenation is also inverted, with higher fluorescence in the oxygenated than deoxygenated state (Fig 3C). In these proteins, Trp40 is more rather than less exposed when the protein is oxygenated. This observation suggests that these mutations yield a novel quaternary conformation, presumably in the oxygenated state, that preferentially binds to both IHP and oxygen.

### 4. Conformational heterogeneity is mediated by ancient features.

How could tetramerization alone immediately confer switching between conformations upon oxygen binding/release? To understand this effect, we examined the phylogenetic age of the key sequence and structural determinants of Hb’s tertiary and quaternary conformational heterogeneity (4, 23). In extant Hb, oxygen binding causes the heme cofactor to become planar, which in turn repositions two histidine residues that bond to the heme – one from “above” on helix E and one from “below” on helix F. This causes helix E and F to move, which in turn changes the position of contiguous secondary elements -- a loop that leads from helix E to helices D and C, and the corner that connects helix F to helix G. Helix C and the FG corner both contribute residues to the tetramerization interface, including Trp40 (on helix C) and the residues it contacts across the interface (on the FG corner). Oxygen binding thereby changes the shape of the interface, causing the two dimers to rotate relative to each other, where they are stabilized by numerous other intersubunit interactions unique to the oxy or deoxy conformations (4, 23, 39).

**Fig. 4.**
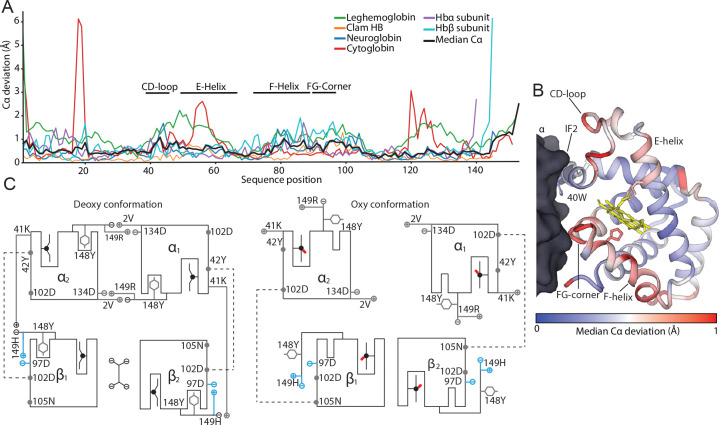
Structural causes of Hb conformational heterogeneity predated the evolution of allostery. **(A)** Deviation in the position of the Cα atom of every residue between aligned oxygenated and deoxygenated X-ray crystal structures of 6 distantly related members of the globin superfamily. Black, median across the 6 pairs. Structural elements important for tertiary and quaternary heterogeneity in human Hb are marked. PDBs for deoxy and oxy structures: clam 4SDH and 1HBI, leghemoglobin 2LH7and 2GDM, cytoglobin 1V5H and 3AG0, neuroglobin 1Q1F and 1W92, human Hbα and Hbβ 4HHB and 2DN1. **(B)** Structure of Anc1 predicted by AlphaFold3, focusing on the secondary elements that surround the heme (yellow) and lead to tetramerization interface IF2. One subunit across IF2 is shown as gray surface and the other as cartoon, colored by the median Cα deviation between oxygenated and deoxygenated X-ray structures across 6 pairs as in panel (A). Trp40 and the histidines that bond to the heme are shown as sticks. **(C)** Reproduction of Pertuz’s classic diagram of electrostatic interactions distinguishing the deoxygenated (left) and oxygenated (right) quaternary conformations, with residues colored by the phylogenetic age of the residues. Residues in grey emerged prior to Ancαβ, residues in blue emerged on the branch leading to Ancβ.

Phylogenetic consideration of prior biochemical work indicates that these features evolved long before Ancαβ and the acquisition of allostery or tetramerization. At the tertiary level, the oxygenation-coupled movement of helices E and F -- and of the regions contiguous to them (FG corner and CD loop) – has been documented in paralogs of Hb, including myoglobin, neuroglobin, cytoglobin, and the blood globins of agnathans (40–45). We also compared the oxygenated vs. deoxygenated X-ray crystal structures of six even more distantly related globin family members (Figs. 4A, 4B, S10). In every case, there are major conformational differences at the CD-loop, E-helix, F-helix, and FG-corner. This finding indicates that oxygen-mediated tertiary movements in these regions have been present since at least the last common ancestor of animals and plants, which occurred ~ 1 billion years prior to the emergence of Hb allosteric regulation (Fig. 4A). This deep ancestry can be explained by the fundamental chemistry of heme – which intrinsically changes planarity when oxygenated -- and the tertiary fold that is common to all globins: in all cases, the heme is coordinated by homologous histidines on homologous helices, both of which lead to the same patch on the surface (Figs 4B, S10).

At the quaternary level, the residues that mediate the distinct contacts that stabilize the oxygenated and deoxygenated conformations in modern Hb are also ancient. Fig 4C reproduces Perutz’s classic diagram of electrostatic interactions that distinguish Hb’s T and R conformations but adding information about the age of the residues. Of 7 interactions that are distinct between the human Hb T and R conformations, 6 involve residues that were all acquired before Ancαβ.

The residues and structural features that mediate the conformational heterogeneity at the heart of Hb allostery are therefore virtually all ancient, substantially predating the evolution of allostery itself. As soon as tetramerization was acquired, the ancient features of tertiary structure that coupled conformational changes to the oxygenation state of the heme propagated into distinct quaternary rotations.

### 5. Structural mechanisms for the evolution of allostery.

Finally, we used molecular dynamics simulations to gain insight into the potential structural mechanisms underlying the evolution of allostery. The simplest hypothesis to explain how the 4 cavity substitutions confer allostery on Anc1 is that they introduced residues that directly facilitate binding of the effector, but this binding site is accessible only when the protein is in its deoxygenated state.

#### Creating an IHP binding site.

**Fig. 5.**
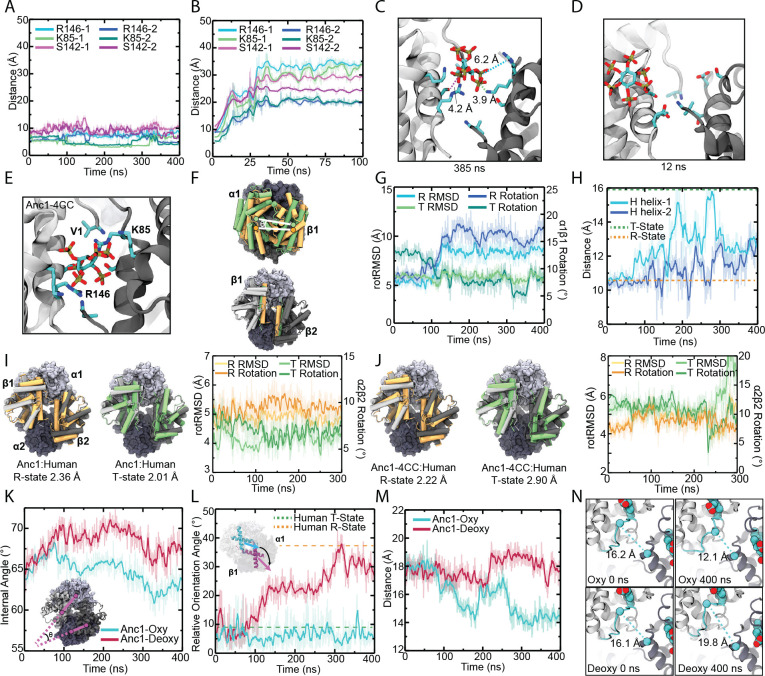
Structural mechanism for the evolution of allostery. Interactions between Anc1–4CC **(A)** or Anc1**(B)** residues and center of mass of the IHP throughout the MD simulation. Distances were measured from terminal oxygen and nitrogen atoms for serine and lysine, and from the C_ζ_ atom for arginine. **(C)** Stable coordination of IHP in the central cavity of Anc1–4CC near the end of the simulation at the indicated timepoint. **(D)** Exit of IHP from the cavity in Anc1 near the beginning at the indicated time. **(E)** Close-up view of the central cavity of the Anc1–4CC-IHP complex after equilibration, showing the symmetry of interactions between the central cavity residues and IHP. **(F)** Schematics of measurements of dimer rotation angle and rotRMSD (top) and interhelical distance (bottom). *Top:* For rotation angle and RMSD, one dimer of the human R-state (1RD) and T-state (2HHB) tetramers were aligned to each other (gray surfaces); the other dimers are shown in orange and green, respectively. Dots show the center of mass of subunits within each dimer; white lines show the vector between the dots, along with the angle of rotation between the T and R vectors. RMSD is for αC atoms between the unaligned dimers. *Bottom:* Distance between the H-helices, defined as the length of the line segment perpendicular to the axis along each helix, measured across both central cavities. **(G)** The change in rotRMSD and rotation angle when Anc1–4CC-IHP at each timepoint in the simulation is aligned to the R or T state. **(H)** Change in helical distance across the trajectory is shown for both pairs of H-helices in the homotetramer Anc1–4cc-IHP. Dotted lines, interhelical distance in human R-state and T-state crystal structures. **(I)** The equilibrated AlphaFold3 model (grey) of Anc1 is shown aligned to the crystal structure of the human R-state (left, orange; PDB 1IRD) and T-state (right, green; PDB 2HHB). Alignment and RMSD are based on Cα atoms. Right, rotRMSD and rotation angle of Anc1 relative to human R-state (yellows) and T-state (greens). **(J)** The equilibrated AlphaFold3 model (grey) of Anc1–4CC is shown aligned to the crystal structure of the human R-state (left, orange) and T-state (right, green). *Right,* rotation angle and rotRMSD of Anc1–4CC relative to human R-state (yellows) and T-state (greens). **(K and L)** Internal rotation angle and relative orientation angle across the trajectories of Anc1-oxy and -deoxy. *Insets*: schematics of these metrics for measuring the rotation between two dimers within a subunit. In (K), the dots indicate the center of mass of the associated subunits within a dimer, the arrows indicate the axis between them, and the angle between those axes for each dimer is the internal rotation angle. In (L), colored arrows indicate the chosen principal axis of the H-helices within a dimer, and the black arrow indicates the rotation required to align the axes from the two dimers to each other. **(M)** Proximity of the CD loop to its neighboring subunit across IF2, measured as the average distance between the Cα of H47 on the CD loop of one subunit and K98 on the FG corner of the other. **(N)** Structural snapshots of the CD loop and heme pocket at 0 and 400 ns for oxygenated (top) and deoxygenated (bottom) Anc1, with the distance between the CD loop and the bottom edge of the heme indicated (Å).

To test this hypothesis, we used AlphaFold3 to generate structural models of Anc1 and Anc1–4CC tetramers containing deoxygenated heme and IHP bound in the central cavity at the same location as in human Hb (PDB:3HXN; fig S11;(46)). Across a 400 ns trajectory, IHP remained stably bound at this site (Fig. 5A). By contrast, IHP was ejected from the cavity of Anc1 within ~20 ns (Fig. 5B).

The four substitutions confer effector binding by making the pocket electrostatically and sterically complementary for IHP. Throughout the Anc1–4CC trajectory, the derived residues Lys85 and Arg146, both of which are positively charged, form stable electrostatic interactions with IHP’s negatively charged phosphate groups, in contrast to the ancestral residues at these sites in Anc1 (glu85 and glu146), which are negatively charged and repel the effector (Fig. 5C &D; fig S12). Ser142 in Anc1–4CC forms transient hydrogen bonds with IHP (Fig. 5A) and removes a steric clash between the effector and the extra methyl group on the ancestral threonine in Anc1 (Fig. 5D). The fourth substitution (r149H at the protein’s C-terminus) relieves a major steric clash with the effector: in the ancestral state, arg149 occupies the central cavity and is stabilized in this location by a salt bridge to Asp134, but substituting this residue to His149 in Anc1–4CC compromises this salt bridge, allowing the C-terminus to shift outward and opening this space for IHP (Fig. 5E). Because the Anc1–4CC tetramer is symmetrical, each derived residue appears twice in the binding pocket – once on each subunit – so each favorable interaction occurs twice in Anc1–4CC, along both sides of IHP (Fig. 5E).

As predicted, IHP binding in Anc1–4CC is preferentially associated with a low-oxygen affinity conformation. We quantified the quaternary similarity of Anc1–4CC to the classic T and R states of human Hb (which have low and high oxygen affinity, respectively) as follows. We aligned one of the dimers of Anc1–4CC to one of the dimers in the X-ray crystal structure of human Hb (in either the T or the R state); we then measured the angle of rotation between the central axis of the other two dimers of Anc1–4CC and that of the human Hb structure, as well as alpha-carbon RMSD between all residues in the second unaligned dimer of the two proteins (Fig. 1F; *top*). We also measured the distance between the H-helices of the two subunits across the central cavity, which is much larger in the human T than R conformation, creating space for IHP to bind (Fig. 1F; *bottom*). In the trajectory with IHP, Anc1–4CC undergoes a notable transition in quaternary structure, becoming much more similar to the human T conformation, and the interhelical distance increases by about 2.5 Å, allowing sufficient space for the effector (Fig. 5G&H). This conformational transition is not observed in the trajectory of Anc1 with IHP (Fig. S13) and is therefore a consequence of IHP binding.

#### Increased oxygen affinity in the absence of IHP.

The 4CC substitutions also improve oxygen affinity in the absence of IHP, which magnifies the allosteric response to the effector when it is present. A simple mechanism to explain this observation would be that the 4CC mutations increase the propensity of the protein to occupy an R-like state when IHP is absent. Corroborating this hypothesis, the quaternary conformation of Anc1–4CC without IHP is considerably more R-like than Anc1 (Fig 5I & J). This change arises because the 4CC substitutions abolish interactions that favor the T-like conformation of Anc1, thus reducing occupancy of the state. Specifically, ancestral residues glu146 and arg149 in Anc1 form strong electrostatic interactions across the central cavity in the T conformation (to the N-terminal amine and Asp 134, respectively); ser85 also hydrogen bonds to either glu146 on the same subunit or His3 on the opposing subunit (fig S14). Substitution of these residues to the derived states Arg146, His149, and Lys85 abolishes these interactions and thus destabilizes the deoxy conformation in Anc1–4CC (fig S15 A). Instead, these derived residues coordinate chloride ions throughout the trajectory, which neutralize the net positive charge they confer in the absence of IHP and facilitate an interaction between the derived residues R149 and K85, which is possible only in the oxygenated conformation (fig S15 A–C).

#### Ancient conformational heterogeneity.

We also used MD to understand the mechanisms underlying the ancient oxygen-dependent conformational heterogeneity of Anc1. Beginning from the T-like equilibrated structure of Anc1 described above, we removed the IHP and initiated 400 ns MD simulations with oxygenated heme. We assessed potential changes in quaternary structure during this trajectory using two metrics of quaternary structure: first, the angle of rotation between the central axes of the two dimers, and second, the angle of rotation between a vector defined by the dominant principle axis of the H-helices in one dimer and that in the other (Fig 5K). The first metric captures the overall orientation of the two axes relative to each other, while the second captures the impact of quaternary rotation on the helices that frame the central cavity. We also initiated a control trajectory from an identical starting point with identical conditions but using deoxygenated heme.

In the deoxy-heme trajectories, the central axis angle increases, and the H-helix rotation angle decreased slightly, as the protein adjusts to the removal of IHP from the central cavity. By contrast, the trajectories with oxygenated heme show a markedly different change in conformation, with the central axis angle declining and the helix rotation angle increasing by ~15 degrees. (Fig. 5L) The difference between oxy and deoxy states goes in the same direction as that between the R and T conformations of human Hb, although the magnitude of difference in the ancestral protein is considerably smaller (Fig 5L; see also fig S16).

As in human Hb, the quaternary changes caused by oxygen binding are associated with tertiary changes near the heme pocket that change the multimerization interface and favor the transition to a new conformation. In the starting deoxy structure, the loop between helices C and D is close to the heme binding site; with oxygenated heme, this loop flips away from the bottom edge of the heme (Fig. 5M&N). These changes alter the network of interactions across the interface (Fig S17). The quaternary rotation of Anc1 coupled to oxygen binding is therefore associated with tertiary changes in the heme pocket and tetramerization surface that are qualitatively similar to those observed in extant Hb.

Taken together, these data provide a structural explanation for the simple acquisition of allostery when central cavity substitutions are introduced into the nonallosteric precursor Anc1. As soon as Trp40 confers tetramerization on Anc1, ancient tertiary heterogeneity is transformed into oxygen-linked quaternary heterogeneity. All that remains is for IHP binding to be acquired at a site preferentially available in the low-oxygen-affinity conformation – precisely what that the 4CC substitutions confer. These same changes also improve oxygen affinity by destabilizing the low-affinity state in the absence of IHP, magnifying the allosteric impact of the effector.

### 6. Mechanisms for inverse allostery.

Finally, we used MD simulations to understand how inverse allostery – increased oxygen affinity upon exposure to IHP – is conferred on Anc1 by single central cavity substitutions. We focused on Anc1-t142S, because this mutant had the strongest inverse allosteric response. We used Alphafold3 to generate an initial structure of Anc1–142S with oxygenated heme, equilibrated it for 100 ns, docked IHP into the structure, and initiated a 300 ns trajectory (46).

**Fig. 6.**
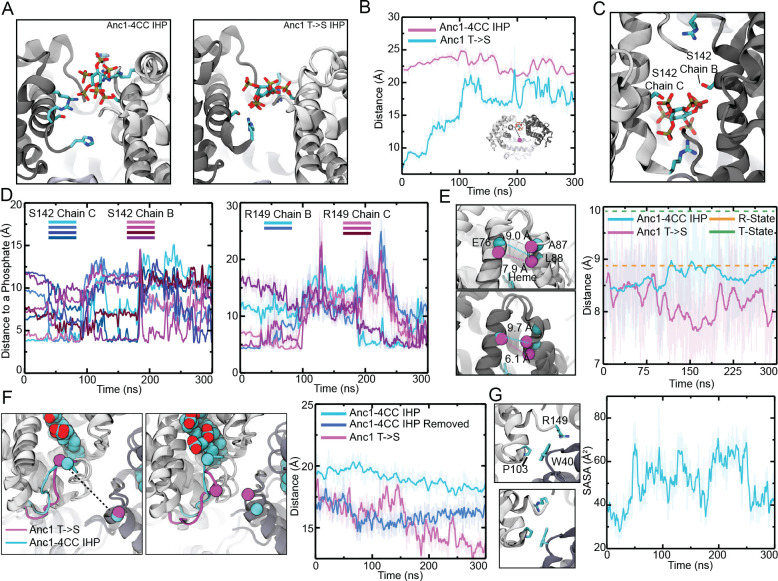
Structural mechanisms of inverted allostery in Anc1-t142S. **(A)** CC residues shown in licorice representation for Anc1–4CC (left) and Anc1- t142S (right), showing the closer association of the IHP in the central cavity and direct association with S142 and the C-terminal R149. **(B)** Distance between the center of mass of the heterotetramer and the center of mass of IHP across the trajectories of Anc1–4CC-IHP and Anc1-t142S. After ~100 ns, an equilibrium is reached, and interaction with S142 keeps IHP lower in the cavity than in Anc1–4CC. *Inset,* schematic of this measurement, with one subunit removed for clarity. **(C)** Close view of the central cavity of the Anc1-t142S model with docked IHP after equilibration. **(D)** Distances between the Oγ of S142 (left) or the C_ζ_ of R149 (right) and phosphates of the IHP are shown throughout the simulation. **(E)** *Left,* measuring the distance between helices E and F across the heme pocket, using the distances between Cα atoms of E76 and L88 (spheres). Top, superposition of Anc1–4CC-IHP and Anc1-t142S at the beginning of the trajectory; bottom, end of the trajectory. *Right,* The average of the this E-F distance across each tetramer’s four subunits is shown at each timepoint in the simulations, along with the same measurement for human R- and T-state crystal structures (dotted lines, PDB 1IRD and 2HHB). **(F)** *Left,* Distance between the CD loop and its neighboring subunit, as described in Fig. 5M, with heme as spheres. Structures from representative frames of each trajectory are shown. *Right,* the average of this distance for the four subunits of each tetramer is shown across the trajectory of Anc1–4CC-IHP and Anc1-t142S. **(G)** The C-terminal carboxyl of R149 is pulled into the central cavity of Anc1-t142S and shield Trp 40, as shown in structures from the beginning and end of the trajectory (*left)*. *Right*, increase in average solvent-accessible surface area of Trp40 residues in this trajectory.

As predicted from the fluorimetry experiments, the Anc1-t42S mutant occupies a different conformation, because it binds IHP in a novel way. Across the entire trajectory of this complex, IHP is stably bound, but at a location ~ 5 Å deeper in the cavity than its canonical position in Anc1–4CC-IHP and in human Hb (Figs. 6A, 6B). IHP is stabilized in this new location by hydrogen-bonds to S142, as well as salt bridges to the ancestral residues arg149 and the N-terminal amine – interactions that are not observed in any other ancestral IHP-bound complex we examined or in the human Hb-IHP crystal structure (Fig 6C & D).

This novel mode of binding IHP is associated with conformational differences that explain the inverse allosteric behavior of this protein. With IHP in this deeper position, the C- and N-termini are pulled into the central cavity of the protein, where they interact with IHP’s phosphate groups as described above (fig S18A–C). Movement of the C-terminal helix is associated with a change in the F helix, which packs against the C-terminal helix and also forms one wall of the heme pocket, moving inward towards the heme by ~1 Å compared to Anc1–4CC (Fig. 6E). This narrowing of the heme pocket is similar to that observed in the R-state of human hemoglobin, which better accommodates the planar oxy-heme than the domed deoxy-heme. In addition, the C/D loop flips away from the bottom edge of the heme, packing in a way that is more similar to the R-like Anc1–4CC than when IHP is bound to Anc1–4CC (Fig. 6F). These observations provide a likely mechanism for the increased oxygen affinity associated with IHP binding by this protein.

Changes also occur at the tetramerization interface, explaining why Anc1–142S displays the inverted fluorimetry pattern that we observed in our experiments. Residues on the C-terminal helix play a major role in packing around Trp40, and repositioning this helix in Anc1142S-IHP abolishes these interactions (Fig. 6G). Trp40 therefore becomes significantly more exposed to solvent across the trajectory in the presence of IHP, consistent with the inverted pattern for this protein in our fluorimetry experiments. Our finding that substitution r149H – the protein’s C-terminal residue -- also confers inverse allostery and the inverted fluorimetry pattern is consistent with the key role of the C-terminal helix in mediating the acquisition these properties.

Taken together, these data show that historical mutations in various combinations can confer on Anc1 IHP binding at multiple locations within the central cavity. The particular location at which this occurs determines whether allostery is acquired in the classical or inverted form.

## DISCUSSION

Our work shows that Ancαβ, the nonallosteric dimer that historically preceded the emergence of the allosteric Hb tetramer, was close to the evolutionary edge of evolving multiple forms of allostery. As few as two historical substitutions are sufficient to confer on it an allosteric response to IHP-- one on the surface to confer tetramerization and another in the central cavity to create an effector binding site that confers positive allosteric regulation. Adding just two more adjusts the location of the optimal binding site for the effector and yields the classic negative allostery observed in extant Hb.

Allostery could evolve by so few substitutions because key structural requirements for allostery were already present in the non-allosteric precursors Ancαβ and Anc1 as biochemical spandrels–by-products of protein structure that were initially functionally inconsequential but became incorporated later into allosteric regulation. Heterogeneity of tertiary structure linked to oxygen binding arises as a by-product of the basic globin fold and the fundamental chemistry of heme (47). Quaternary heterogeneity is a by-product of this tertiary heterogeneity and the fortuitous fact that the tetramerization interface evolved at a surface affected by the tertiary heterogeneity. This quaternary rotation affects conformation at the tetramer’s central cavity, providing a potential effector-binding site that is differentially available depending upon oxygen binding.

Although Hb is a singular example, many other allosteric proteins may have evolved suddenly from nonallosteric precursors that fortuitously possessed many of allostery’s prerequisites. Virtually all proteins occupy multiple conformations rather than a single rigid structure (48–54). These conformations typically differ in activity, a corollary of conformational selection and induced fit mechanisms of ligand/substrate binding (51, 55–59). And conformational differences that affect a protein’s active site are typically associated with changes elsewhere in the protein, simply because a protein’s fold involves covalent linkages and dense packing between elements (60–63). If mutations confer binding to a potential effector at a site that is conformationally associated with changes at the active site, then allostery will be immediately acquired. And numerous studies show that binding to small molecules or proteins can often be conferred by one or a few mutations on a protein’s surface (11, 12, 64). Consistent with this view, several protein engineering studies and mutational scans have shown that allostery can be conferred on nonallosteric present-day proteins by single point mutations or insertions of effector-binding sites (12, 65–68). Moreover, proteins that have no known endogenous allosteric regulators are often “latently allosteric:” they can be regulated by drugs that bind to surface sites conformationally associated with the active site, indicating that they fortuitously posses all the prerequsites for allostery without any biological relevance (9, 61, 69–71).

Hb was on the evolutionary edge of at least two forms of allostery – increasing or reducing oxygen affinity upon IHP binding – depending on the particular subset of the 4 central cavity substitutions introduced first, which determine the precise location at which the effector is bound. Hb’s potential to evolve multiple forms of allostery arises because each binding site is associated with slightly different quaternary ensembles around the central cavity, and these have different associations with tertiary structure at the active site. This also appears to be a generalizable observation: historical reconstructions and protein engineering/mutation studies show that the functional impact of effector binding in an allosteric protein can often be reversed by one or a few amino acid changes (15–17).

Our results suggest the contingent acquisition of prerequisites for allostery rather than long paths driven by selection. The conformational heterogeneity present in Hb’s evolutionary precursors could not have been driven by selection for allostery, because it is not sufficient to confer even a weak allosteric response. If tetramerization had not evolved, or if it had evolved at a different patch that is not conformationally linked to the remodeling of the heme binding site, then allostery could not have evolved by the substitutions in the central cavity. It has also been proposed that conformational heterogeneity may evolve because of selection on proteins to be adaptable in the face of fluctuating biological environments (40, 41). In hemoglobin, this does not appear to be the case: tertiary heterogeneity, which is extremely ancient, is a simple by-product of the protein’s architecture, and it has no known functional consequences. Our data do not resolve the order in which the key substitutions occurred, but contingency would have pertained whether the substitution at IF2 or those at the central cavity evolved first: in either case, a fully nonallosteric precursor would have been as little as one substitution away from acquiring allostery.

Across the protein universe, allostery is strongly enriched among isologous multimers (34, 49, 72, 73). Our findings suggest that isology facilitates the mutational accessibility of allostery in Hb in at least three ways, which are likely to be general. First, isology facilitates the acquisition of effector binding. In an isologous complex, a potential binding site for an effector that involves multiple subunits is also symmetric; a single substitution that facilitates effector binding at such a site will therefore occur twice, doubling its energetic contribution to binding and exponentially increasing its effect on affinity (34). Second, isology amplifies the spatial magnitude of conformational change from small tertiary changes – such as those at IF2 – to much larger quaternary differences, such as those in the central cavity. Third, isology enlarges the mutational target size of sites at which effector binding can regulate a protein’s activity. In a multimer that assembles via an isologous interface that is conformationally heterogeneous and coupled to the active site, this heterogeneity will propagate to every part of the oligomer surface that comprises multiple subunits.

Isologous multimers, including Hb, can themselves evolve from lower-order forms by single substitutions(24, 34, 74). Our data suggest that such complexes are often and immediately on the edge of evolving allostery. The structural and functional form of allostery that emerges next depends on the particular mutations that happen to bring proteins to the edge, and those that push them over into the not-so-mysterious state of allostery.

## METHODS

### Sequence data, alignment phylogeny, and ancestral sequence reconstruction.

Sequence data, alignment phylogeny, and ancestral sequence reconstruction. The reconstructed ancestral sequences used here are the same as those reported previously (24). Briefly, 177 amino acid sequences of hemoglobin and related paralogs were collected and aligned. The maximum likelihood (ML) phylogenetic tree was inferred using the AIC best-fit model, LG+G+F (75). The phylogeny was rooted using as outgroups neuroglobin and globin X, which are found in both deuterostomes and protostomes and diverged prior to the gene duplications that produced vertebrate myoglobin and the hemoglobin subunits. Ancestral sequence reconstruction was performed using the empirical Bayes method, given the alignment, ML phylogeny, ML branch lengths, and ML model parameters (76). Reconstructed ancestors that were used in this study have been deposited previously in GenBank (IDs MT079112, MT079113, MT079114, MT079115).

For the set of historical mutations *Central Cavity (CC)*, all sites at central cavity that were substituted between Ancαβ and Ancβ are changed to the derived state found in Ancβ; the mutations introduced are s85K, t142S, e146R, and r149H. The substitution q40W, refers to a single substitution that occurs on the branch leading to Ancβ that is sufficient to confer tetramerization on the ancestral background Anc1–4CC is the union of the single substitution q40W and the set *Central Cavity*.

### Recombinant protein expression

Coding sequences for reconstructed ancestral proteins were optimized for expression in Escherichia coli using IDT Codon Optimization and synthesized de novo as gBlocks (IDT). Coding sequences were cloned by Gibson assembly into vector pLIC under control of a T7 polymerase promoter (77). For co- expression of Ancα+Ancβ, a polycistronic operon was constructed under control of a T7 promoter and separated by a spacer containing a stop codon and ribosome binding site, as described in (78).

JM109 (DE3) Esherichia coli cells (New England Biolabs) were heat-shock transformed and plated onto Luria broth (LB) containing 50 ug/mL carbenicillin. For the starter culture, a single colony was inoculated into 50 mL of LB with 1:1000 dilution of working- stock carbenicillin and grown overnight. 5 mL of the starter culture were inoculated into a larger 500-mL terrific broth (TB) mixture containing the appropriate antibiotic concentration. Cells were grown at 37° C and shaken at 225 rpm in an incubator until they reached an optical density at 600 nm of 0.6–0.8.

For expression of single globin proteins, 100 uM of isopropyl-β-D-1- thiogalactopyranoside (IPTG) and 25 mg/500 mL of hemin were added to each culture. Expression of single proteins in culture were done overnight at 22° C. Cells were collected by centrifugation at 4,000g and stored at −80° C until protein purification. Coexpressed proteins were induced using 500 mM IPTG expression with 25 mg/500 mL hemin for 4 hours at 37°C. Cells were collected by centrifugation at 4,000g, immediately followed by purification.

Human hemoglobin was bought commercially (Sigma-Aldridge) and resuspended in PBS.

### Protein purification by ion exchange

Purification of singly expressed ancestral hemoglobin proteins were done as previously described (34). All singly expressed proteins (all ancestral globins except Ancα+Ancβ) were purified using ion exchange chromatography, with variation in buffer pH. All buffers were vacuum filtered through a 0.2 μM PFTE membrane (Omnipore). After expression, cells were resuspended in 30 mL of 50 mM Tris-Base (pH 6.88). The resuspended cells were placed in a 10 mL falcon tube and lysed using a FB505 sonicator (1s on/off for three cycles, each 1 minute). The lysate was saturated with CO, transferred to a 30 mL round bottom tube, and centrifuged at 20,000g for 60 minutes to separate supernatant from non-soluble cell debris. The supernatant was collected and syringe-filtered using HPX Millex Durapore filters (Millipore) to further remove debris. A HiTrap SP cation exchange (GE) column was attached to an FPLC system (Biorad) and equilibrated in 50 mM Tris-Base (pH 6.88). The lysate was passed over the column. 50 mL of 50 mM Trise-Base (pH 6.88) was run through the SP column to remove weakly bound non-target soluble products. Elution of bound ancestral Hbs was performed with 100-mL gradient of 50mM Tris-Base 1 M NaCl (pH 6.88) buffer which was run through the column from 0% to 100%. For the construct Anc1–4CC, all buffer pH for purification were 6.47. 2 mL fractions were captured during the gradient process, all fractions containing red eluant were put into an Amicon ultra-15 tube and concentrated by centrifugation at 4,000g to a final volume of 1 mL. For additional purification, concentrated sample was injected into a HiPrep 16/60 Sephacryl S-100 HR size exclusion chromatography (SEC) column. The column was equilibrated in phosphate buffered saline (PBS) at pH 7.4. Purified ancestral globins elute at different volumes depending on the protein’s complex stoichiometry: 48–52 for tetramers, 56–60 for dimers, and 65–67 for monomers. The purified proteins were concentrated as mentioned above and then flash frozen with liquid nitrogen.

Protein purification by zinc affinity chromatography. Coexpressed proteins Ancα + Ancβ were purified using zinc-affinity chromatography, which was performed using a HisTrap metal affinity column (GE) on a Biorad NGC Quest. Nickle ions were stripped from the column (buffer 100 mM EDTA, 100 mM NaCl, 20 mM TRIS, pH 8.0), followed by five column volumes of water. To attach zinc to the column, 0.1 M ZnSO4 was passed over until conductance was stable, approximately 5 column volumes, followed by five column volumes of water. After expression, cells were resuspended in a 50 mL lysis buffer (20 mM Tris, 150 mM Nacl, 10% glycerol (v/v), 1mM BME, 0.05% Tween-20, and 1 Roche Protease EDTA-free inhibitor tablet, pH 7.40), sonicated as described above, and the lysate passed through the prepared column. To remove non-specifically bound protein, the column was washed with 50 mL of lysis buffer. Bound protein was then eluted across a gradient of imidazole concentrations (0 to 500 mM) in a total of 100 mL elution buffer (20 mM Tris, 150 mM NaCl, 500 mM imidazole, 10% glycerol, and 1 mM BME, pH 7.4). 1 mL fractions were collected. The fraction corresponding to the second peak of UV absorbance at 280 nm has a visible red color and was collected and concentrated as described above. The concentrated solution was injected into a Biorad ENrich 650 10 × 300 columns for additional purification and eluted in PBS buffer.

### Measuring oxygen affinity and allostery

Purified proteins were deoxygenated using 10 mg/mL sodium dithionite and immediately desalted via a PD-10 column (GE Healthcare) equilibrated with 25 mL of 10 mM HEPES, 0.5 mM EDTA (pH 7.4). Eluted proteins were concentrated using Amicon Ultra-4 centrifugal filters (Millipore).

Equilibrium oxygen-binding assays were conducted at 25°C using a Blood Oxygen Binding System (Loligo Systems) with 0.1 mM protein (heme concentration) dialyzed in 100 mM HEPES, 0.5 mM EDTA buffer. Protein solutions were sequentially equilibrated at 3–5 oxygen tensions (PO_2_), achieving 30–70% saturation, while continuously monitoring absorbance at 430 nm (deoxy peak) and 421 nm (oxy/deoxy isosbestic point). Fractional saturation was plotted against PO_2_, and the Hill equation was fitted to each dataset using OriginPro 2016 to estimate P_50_ (PO_2_ at half-saturation) and the Hill coefficient (n_50_, slope at half-saturation). Confidence intervals (95%) were calculated by multiplying the standard error of the mean from replicate experiments by 1.96.

To assess potential allosteric regulation by organic phosphate effectors, assays were conducted under three conditions: without effectors (stripped), with 0.5 mM IHP, and with 0.5 mM ATP. Most experiments were performed with IHP due to its stronger allosteric effect compared to ATP and their qualitatively similar modulation of Hb-O_2_ affinity (19, 20). Although IHP may not have been the physiological effector in ancestral organisms, it has been shown to allosterically regulate hemoglobins across major vertebrate lineages, whereas effectors such as 2,3-bisphosphoglycerate (BPG), ATP, and GTP exhibit lineage-specific effects. Thus, IHP serves as a general polyanion for assessing the allosteric capacity of ancestral Hb, a well-established approach in hemoglobin studies (24, 35).

### Fluorescence emission scan

Purified proteins were deoxygenated with 10 μM sodium dithionite to remove oxidized hemoglobin, then desalted using a PD-10 column (Cytiva) equilibrated with PBS. If necessary, eluted proteins were concentrated using Amicon Ultra-15 filters prewashed with PBS.

To evaluate conformational states as a function of oxygenation, proteins were aliquoted into two 150 μL samples at 150 μM concentration. Buffer-only aliquots were prepared for fluorescence background correction. Oxygenated hemoglobin samples were verified by UV-Vis spectroscopy, confirming the characteristic double peak at 480 nm. Samples were transferred into a sealed sub-micro quart fuorometer cell (Starna Cells, Inc.). Fluorescence scans were performed using a Fluorolog-3 (Horiba) with excitation at 280 nm and emission recorded from 307–500 nm in 1 nm increments. Slit widths for excitation and emission ranged from 4–7 nm. To mitigate inner-filter effects of hemoglobin, a front-facing mirror setup was used, an established approach for Hb fluorescence studies (79).

For deoxygenated samples, an aliquot was incubated in a custom anaerobic chamber saturated with nitrogen for two hours, with periodic resuspension to facilitate oxygen exchange. At the time of measurement, the sample was transferred to the same sealed sub-micro quartz fluorometer cell used in oxygenation condition. Fluorescence scans were conducted immediately after chamber removal.

### Molecular Dynamics Simulations

Models of the Anc1 and Anc2 hemoglobin were generated using the AlphaFold3 web server (46). Multimeric predictions were carried out to model the quaternary structure by including four copies of each monomer, and heme cofactors were included using the ligand modeling capabilities of AlphaFold3. Model confidence was assessed via the predicted template modeling (pTM) and interface predicted template modeling (ipTM) scores. Anc1 and Anc2 exhibited pTM scores of 0.96 and 0.92, respectively, and ipTM scores of 0.95 ad 0.91, respectively, indicating a high degree of confidence in the predictions of both the tertiary and quaternary structures.

The VMD psfgen, solvate, and autoionize plugins were used to build all systems, and all analysis was done using VMD with in-house Tcl scripts or MDAnalysis (80, 81). Water boxes were made large enough to ensure no interactions occurred between periodic images. For both systems, Cl^−^ was added to neutralize charge. The *N*-termini of all proteins was capped with −NH^3+^ and the *C*-termini with a −COO^−^ carboxylate group. Simulations were run using NAMD 2.14^4^ with the CHARMM36^5^ force field and the TIP3P explicit model of water (82, 83). A cutoff of 12 Å was used for van der Waals interactions, and long-range electrostatic forces were computed using the Particle Mesh Ewald method with a grid point density of >1 Å−3. The SHAKE algorithm was used with a timestep of 2 fs. The NpT ensemble was used at 1 atm with a hybrid Nosé-Hoover Langevin piston method, a 200-fs decay period, and with a 100-fs damping time constant. Temperature was set to 300 K. All systems were minimized for 2000 steps, followed by 100 ps of simulation with hemes and backbone atoms constrained, after which constraints were lifted for production MD.

Previous experiments have shown that Cl^-^ can act as an allosteric effector of hemoglobin (84, 85). Because of this, and because the Cl^-^ is necessary to neutralize the excess negative charge of the overall system, the collective variables module in NAMD was used in simulations without IHP to prevent Cl^-^ binding to interfacial regions. A harmonic potential of 100 kcal/mol was applied to any Cl^-^ that drifts within 15 Å of any residue at the α1α2 and β1β2 interfaces. Beyond this cutoff, no potential was applied. This approach permits a charge neutral system, without affecting the allosteric dynamics of the protein in an unintended fashion. Initial equilibration simulations of Anc1 and Anc1–4CC were initiated from this setup, with no further modifications.

Systems with IHP were prepared by first aligning the H-helices of the Anc1 or Anc2 beta domains to the H-helices of the beta domains of the human hemoglobin bound to IHP (PDB code 3HXN). The IHP molecule was then saved and combined with the Anc1 or Anc2 structure, with subsequent solvation and ionization. Parameters for IHP were generated using the CGenFF web server, which resulted in a param penalty of 6.5 and a charge penalty of 7.5, indicating high quality predictions (86).

To initiate simulations after removing IHP, the structure of the protein and hemes, without IHP, was saved from the final frame of the Anc1–4CC with IHP simulation. This was solvated, ionized, and minimized as above. VMD’s mutate plugin was used on the saved structure, prior to solvation, to change the sequence to Anc1, which was then solvated, ionized, and minimized as above. These two systems were used to initiate production MD for Anc1-deoxy and Anc1–4CC-deoxy starting from a putative T-like state. To prepare identical systems in the oxy state, the same saved structure from the Anc1–4CC with IHP simulation as above was used but with the PLO2 patch added to the heme molecules during psf generation, followed by solvation and ionization as above. This resulted in a total of four IHP-removed systems: Anc1–4CC-oxy, Anc1–4CC-deoxy, Anc1-oxy, and Anc1-deoxy. Simulations were run identically as in the equilibration simulations as described above.

To prepare the Anc1 T->S system, an initial structure was generated using AlphaFold3, solvated, and ionized as described above, using the PLO2 patch during psf generation to add O2 ligands to the heme molecules. This structure was used to run a 100 ns equilibrium simulation using identical parameters as above. Hemoglobin in the final frame of this simulation was saved and used to dock IHP into. Hydrogens were added, Gasteiger charges were calculated, and atom type and torsion groups were assigned for IHP using autodocktools. AutoDock Vina^9^ was then run with the entire protein used as a search space. Four of the top five lowest energy docked structures were found in the central cavity (87). The second lowest energy structure (−6.3 kcal/mol) was selected for further simulation and saved as a structure together with the hemoglobin as the starting point for simulation (Fig. 6 A). As a control, VMD’s mutate plugin was used on this structure to mutate the sequence of hemoglobin to Anc1. Both structures were solvated and ionized as above, and simulations were run identically as in the equilibration simulations as described above.

## Supplementary Material

Supplement 1
