# Supplementary material for: Origins of allostery in vertebrate hemoglobin evolution
Source: bioRxiv. 2026 May 26:2026.05.25.727495. Preprint. [Version 1] doi: 10.64898/2026.05.25.727495 (PMC13232183; doi:10.64898/2026.05.25.727495)
Supplement: Supplement 1 [file media-1.pdf]

Supplementary Materials for

Origins of allostery in vertebrate hemoglobin evolution

Carlos R. Cortez-Romero *et al.*

Corresponding author: Joseph W. Thornton, joet1@uchicago.edu

This PDF includes:

Figs. S1 – 18

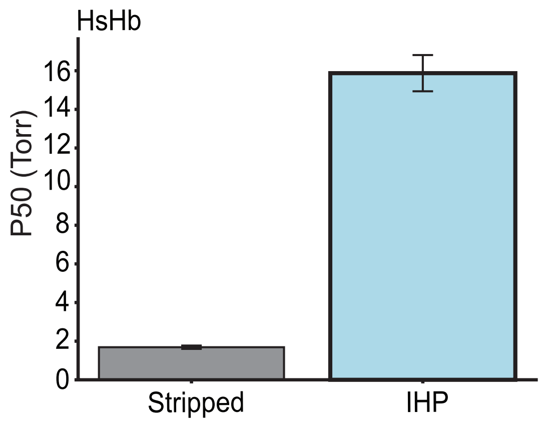

Fig S1. Oxygen affinity of human hemoglobin in the presence and absence of allosteric effector. IHP was added at 500 μM. Error bars show standard error of measurement for 3 replicates.

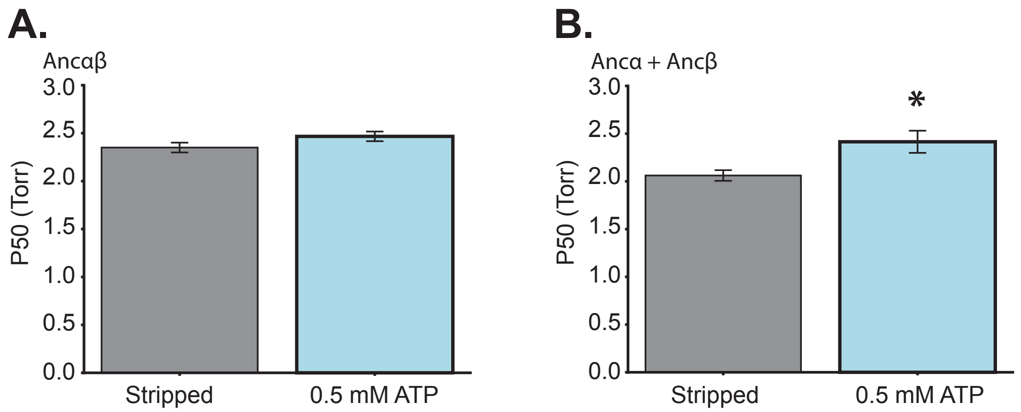

Fig S2. Allosteric response of ancestral proteins to ATP. (A) Bar graph of oxygen affinity of Ancαβ in the presence and absence of ATP. In grey, stripped condition, where no IHP is in solution. In blue, IHP condition, where 500 μM of ATP is added to solution. Error bars, standard error of measurement, n = 3. (B) Bar graph of oxygen affinity of Ancα + Ancβ. Stars represent FDR <0.05 between conditions via Welch's two-sample t-test with Benjamini-Hochberg procedure for multiple testing.

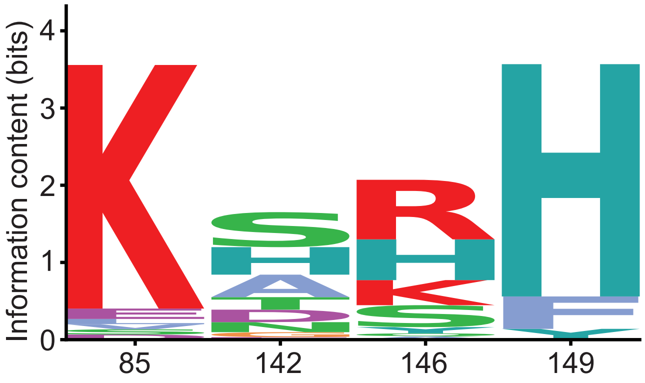

Fig S3. Conservation of central cavity positions in extant hemoglobins. Logo plot of per-position information content (bits) calculated from a multiple sequence alignment of vertebrate hemoglobin β-subunit sequences at the four central cavity residue positions (85, 142, 146, and 149) that substitution fro Anc_1 to $Anc_{1\text{-}4CC}$. Maximum information content is 4.32 bits (invariant position). High information content at these positions indicates strong conservation of the derived residues across extant vertebrate hemoglobins.

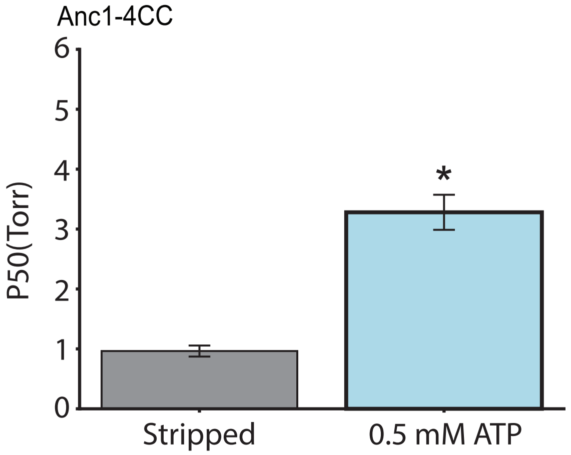

Fig S4. Allosteric response to ATP in Anc1-4CC. (A) Bar graph of oxygen affinity of Ancαβ in the presence and absence of ATP. In grey, stripped condition, where no ATP is in solution. In blue, IHP condition, where 500 μM of ATP is added to solution. Error bars represent standard error of measurement, n = 3. Stars represent FDR <0.05 between conditions via Welch's two-sample t-test and Benjamini-Hochberg FDR procedure.

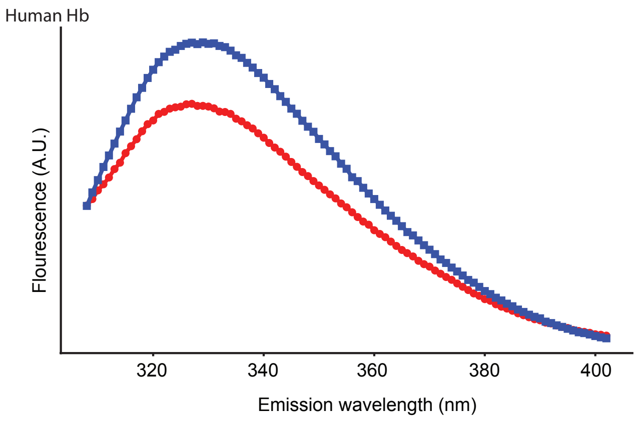

Fig S5. Conformational heterogeneity of human Hb. Fluorescence emissions scans of Human Hb when excited at 280 nm. In red, protein is oxygenated; in blue, deoxygenated. Error bars represent standard error of measurement, n = 10

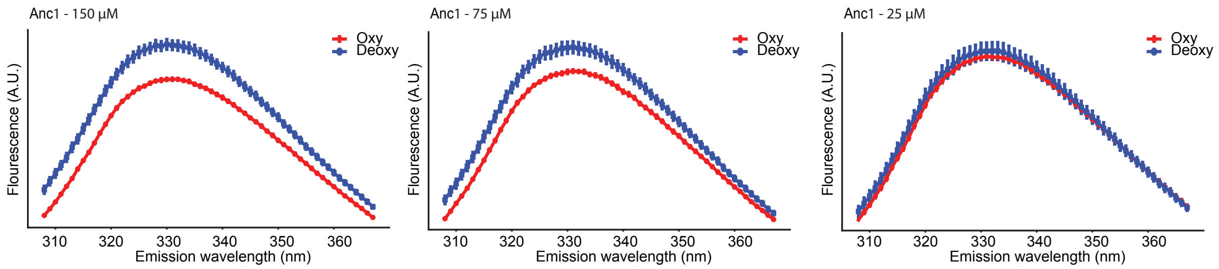

Fig S6. Concentration-dependent fluorescence signal of conformational change in Anc1 indicates tetramer-dependence. Fluorescence emissions scans of Anc1-4CC when excited at 280 nm, across three concentrations. In red, protein is oxygenated; blue, deoxygenated. Error bars represent standard error of measurement, n = 10. As expected, the difference between conditions increases with concentration, because occupancy of the tetrameric stoichiometry increases with concentration, and the quaternary heterogeneity requires tetramerization.

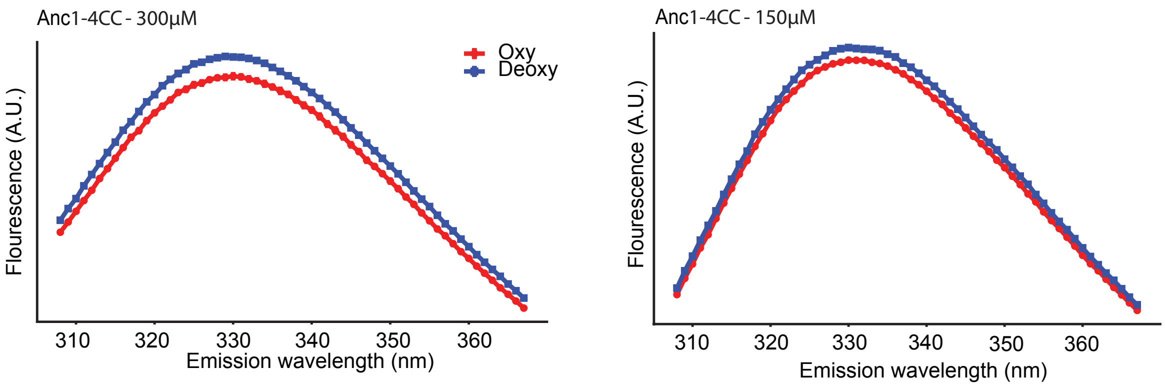

Fig S7. Concentration-dependent fluorescence signal of conformational change in Anc1-4CC indicates tetramer-dependence. Fluorescence emissions scans of Anc1-4CC when excited at 280 nm, at two concentrations. In red, protein is oxygenated; blue, deoxygenated. Error bars represent standard error of measurement, n = 10.

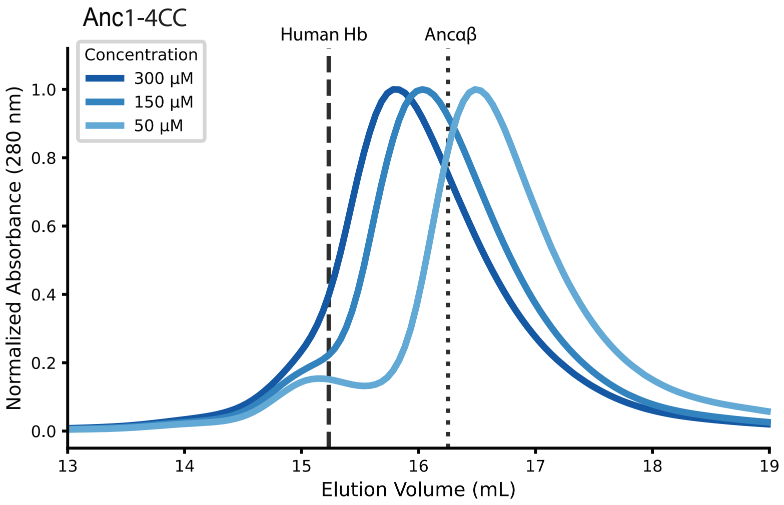

Fig S8. Size exclusion chromatography elution of Anc1-4CC indicates increased tetramer assembly at higher concentrations. Elution traces of size exclusion chromatography at three protein concentrations. Lines at the elution peaks of Human Hb, a known tetramer, and Ancαβ, a known, dimer shown are shown as reference.

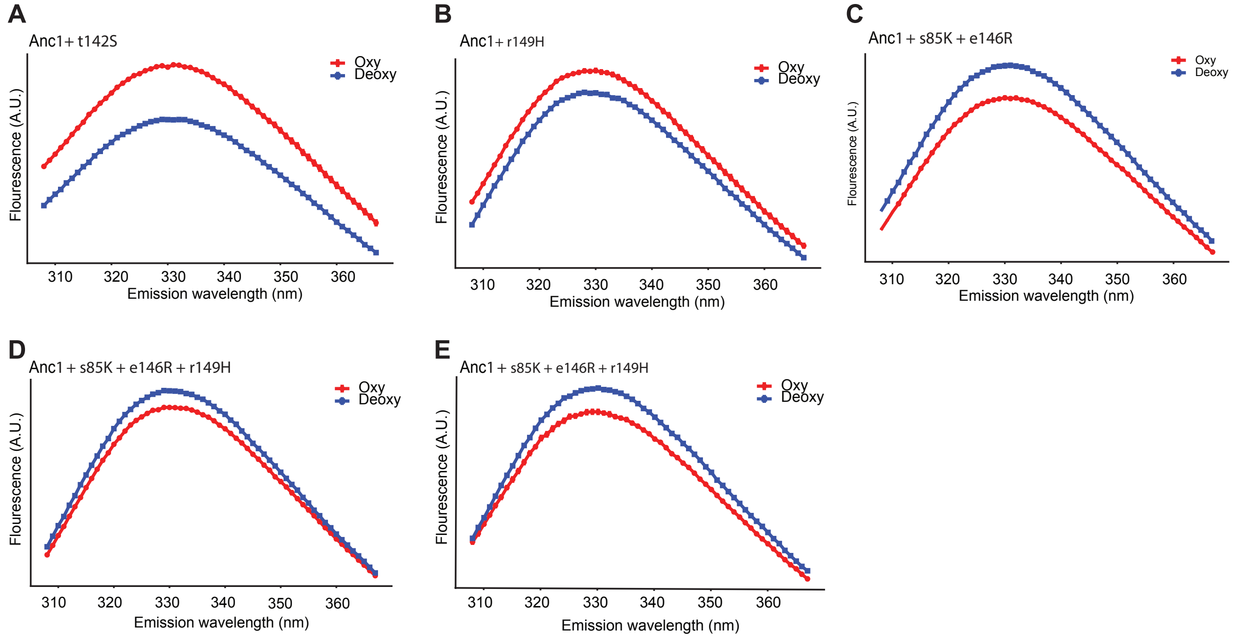

Fig S9. Fluorescence signal of conoformational change Anc1 mutants. For details of representation, see figs. S5-S7. Error bars, standard error of measurement, n = 10.

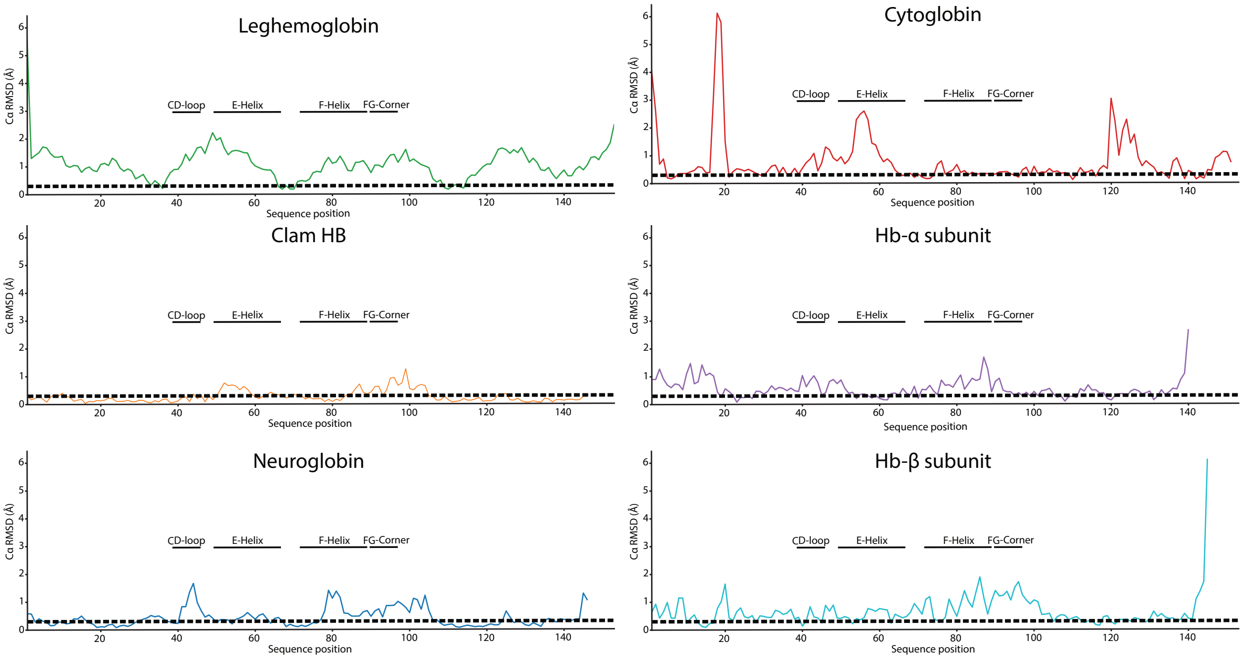

Fig S10. Individual per-residue Cα deviation between oxygenated and deoxygenated crystal structures of globin proteins. Each color represents a distinct globin protein family member: leghemoglobin (green), clam hemoglobin (red), neuroglobin (dark blue), cytoglobin (teal), Hb α subunit (purple), Hb β subunit (cyan). The distance between Cα atoms of oxy and deoxy structures are plotted across the length of the protein. PDB IDs are in the legend for main text Fig. 4.

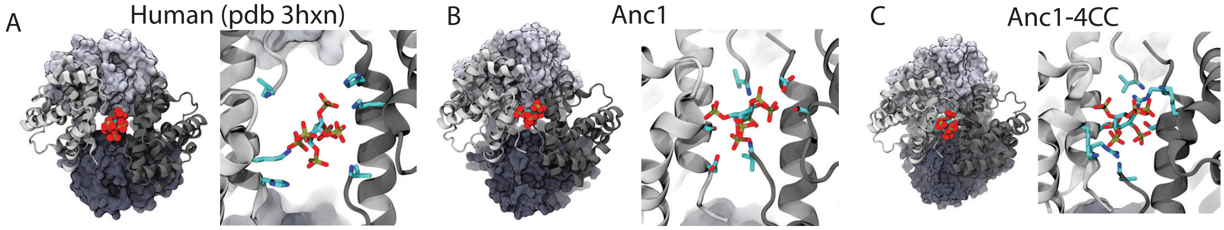

Fig S11. IHP binding cavity is conserved between Human Hb and ancestral hemoglobins. (A) Surface representation (left) and close-up view of the central cavity (right) of Human Hb (PDB: 3HXN) with IHP and key cavity-lining residues shown as cyan sticks. Orange, phosphate atoms; red, oxygen; blue, nitrogen. (B) Equivalent views for Anc1. (C) Equivalent views for Anc1-4CC. Structural models were generated by AlphaFold3 and docked with IHP as described in Materials and Methods.

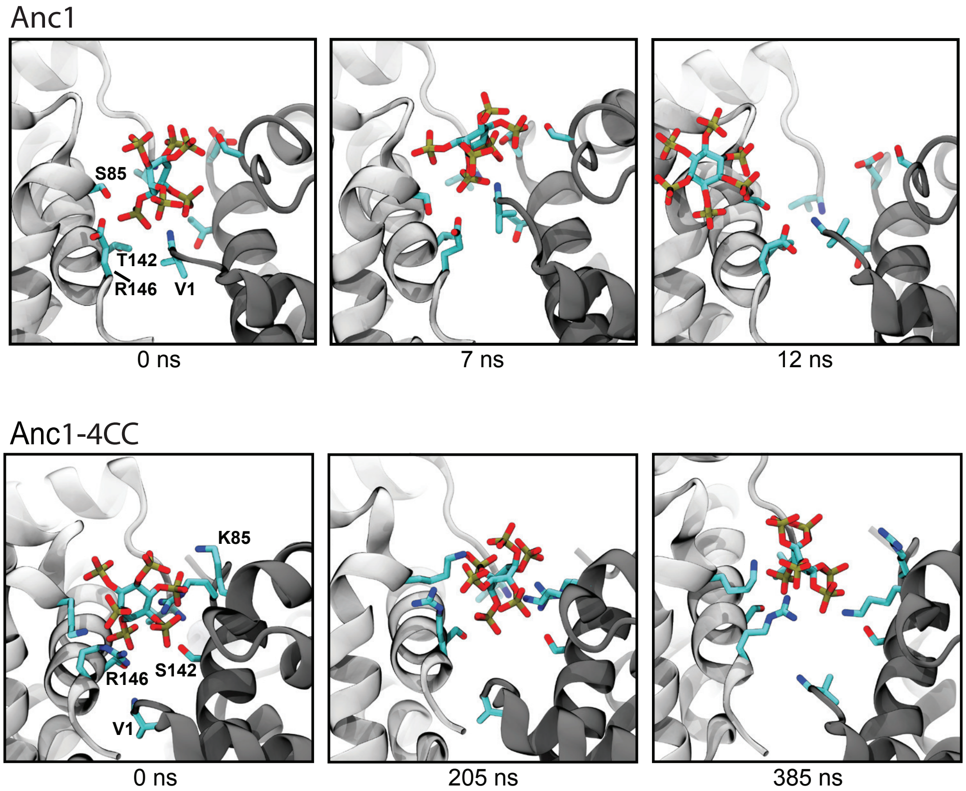

Fig S12. Structural view of IHP binding in Anc1 and Anc1-4CC. Snapshots from MD simulations showing the central cavity residues interacting with IHP (shown in stick representation, cyan with orange phosphate atoms) at the indicated time points. (Top) Anc1 at 0, 7, and 12 ns showing residues S85, T142, R146, and the Val1 N-terminus in cyan. (Bottom) Anc1-4CC at 0, 205, and 385 ns showing the equivalent positions K85, S142, R146, and Val1. IHP rapidly dissociates from the Anc1 central cavity, while Anc1-4CC maintains persistent contacts throughout the simulation.

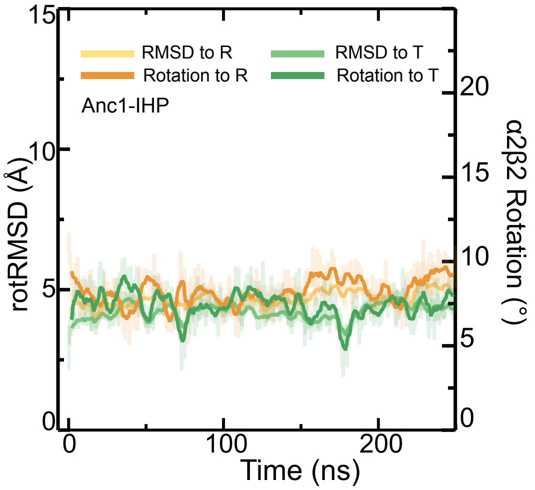

Fig S13. Anc1 does not undergo the R-to-T quaternary transition in the presence of IHP. Rotational RMSD (rotRMSD, Å, left axis) and dimer rotation angle (right vertical axis) of Anc1+IHP relative to human Hb in the oxygenated R or deoxygenated T conformation. The rotation angle represents the angle between the central axes of the unaligned dimers ($\alpha_2\beta_2$) when two tetramers are aligned using the alpha-carbons in the other dimer ($\alpha_1\beta_1$).

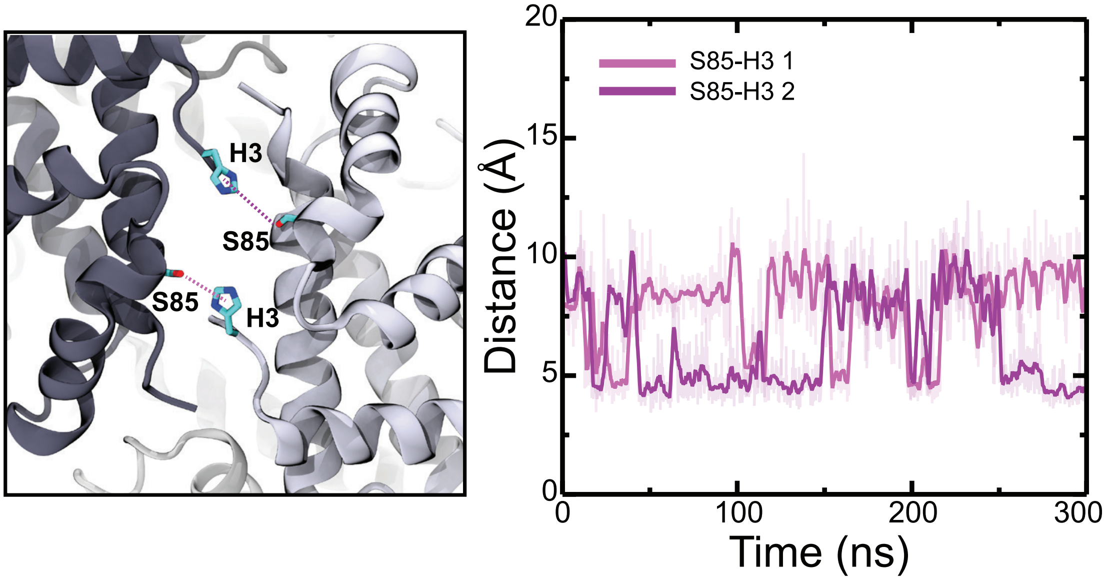

Fig S14. Ser85 makes contacts with His3 across the central cavity in Anc1. (Left) Representative structural snapshot from MD simulation showing S85 (cyan) on one subunit forming contacts (dashed pink lines) with residues in H3 of the opposing subunit. (Right) Distance (Å) between S85 and His3 contact atoms, measured in both isologous iterations across the central cavity.

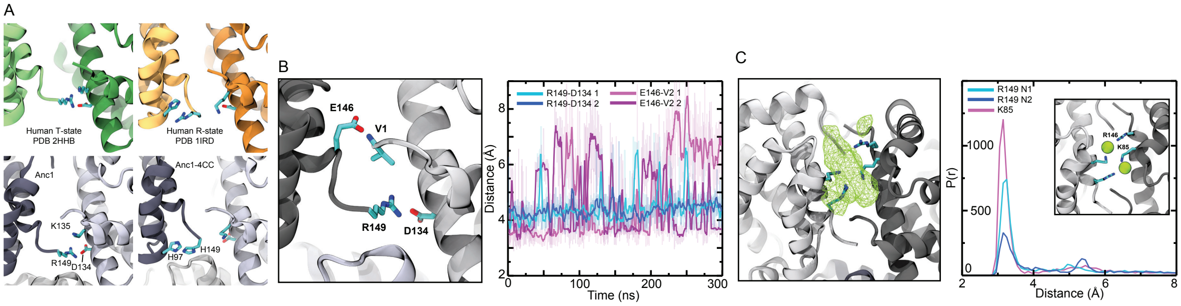

Fig S15. Derived central cavity residues alter the electrostatic environment of central cavity. (A) Structural comparison of the central cavity region across Human T-state (PDB: 2HHB), Human R-state (PDB: 1IRD), Anc1, and Anc1-4CC, highlighting the positions of key residues K135, D134, H149, and R149. (B) Structural view (left) of the Anc1 central cavity without IHP showing E146, R149, D134, and Val1 (V1) in stick representation, with distance traces (right) for R149–D134 (cyan) and E146–V2 (pink) contact pairs across the central cavity. (C) *Left:* Surface representation of Anc1-4CC showing the electrostatic environment of the central cavity and unoccupied cavity (green mesh). *Right:* pairwise distance probability distribution P(r) (right) computed for R149 N1, R149 N2, and K85 contact distances (Å) from MD simulation; inset shows structure of the measured atoms.

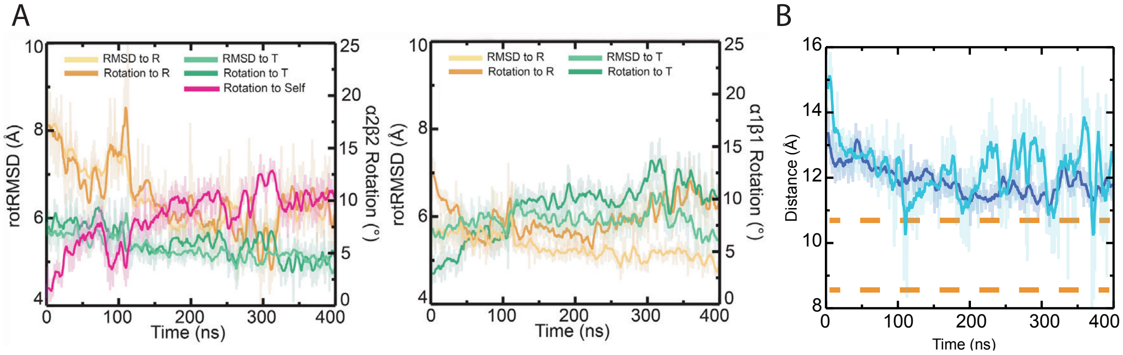

Fig S16. Oxygen dependent conformational change in Anc1. (A) For oxygenated Anc1, the rotational RMSD (rotRMSD, Å, left axis) and dimer rotation angle (right vertical axis) are shown relative to human Hb in the oxygenated R or deoxygenated T conformation. The rotation angle represents the angle between the central axes of the unaligned dimers ($\alpha_2\beta_2$) when two tetramers are aligned using the alpha-carbons in the other dimer ($\alpha_1\beta_1$). (B) Distance between H helices across the central cavity (Å) for oxygenated Anc1 over time; dashed orange line indicates the T-state reference distance.

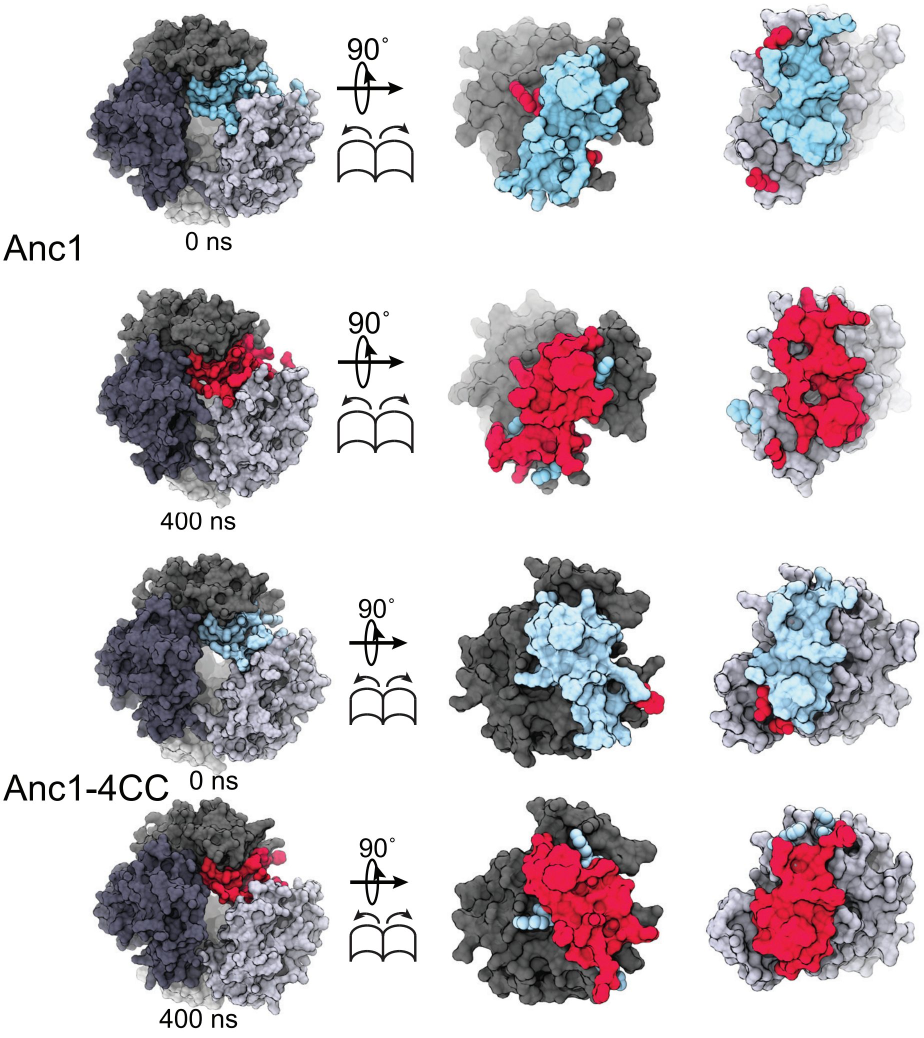

Fig S17. Changes in IF2 surface in Anc1 and Anc1-4CC caused by oxygen-binding. Surface representations of the IF2 interface in Anc1 (top two rows) and Anc1-4CC (bottom two rows). Buried surface residues are colored blue at 0 ns and red at 400 ns in the trajectory with oxygenated heme. Left, intact tetramer; right; tetramer split into its component dimers across IF2. To facilitate comparison, the buried surface at both timepoints is shown in both representations, but with a different timepoint in the top layer in the upper and lower representations.

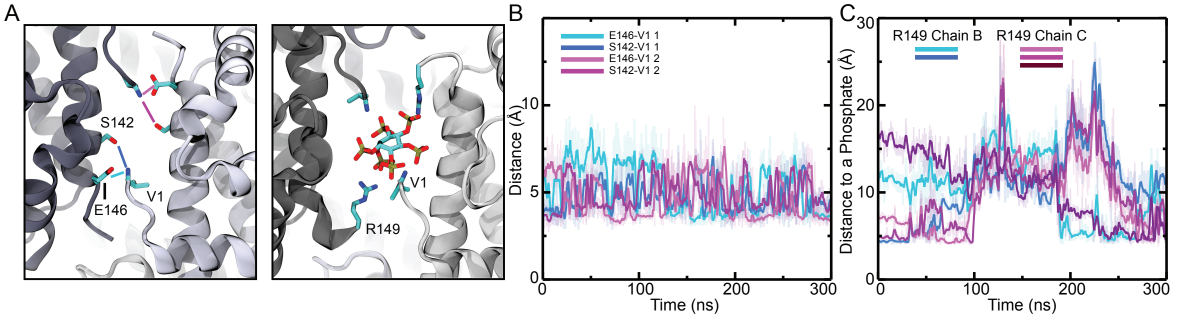

Fig S18. IHP coordination by Anc1-t142S. (A) Structural views of the Anc1-t142S central cavity, without IHP (left) and with IHP (right). IHP is shown as cyan sticks with orange phosphate atoms. One example frame from each trajectory is shown. Colored lines show distances plotted in panel B. (B) When IHP is absent, stable contacts are formed between Val1 (V1) and S142 or E146 residues; distances between these residues are plotted across the trajectory. (C) When IHP is present, it interacts with R149. Distance from R149 residues (on the two chains in the central cavity where IHP binds) to the nearest IHP phosphate oxygen are plotted.
